# Optimization of the extraction and purification of *Corydalis yanhusuo W.T. Wang* based on the Q-marker uniform design method

**DOI:** 10.1186/s13065-020-00666-6

**Published:** 2020-02-11

**Authors:** Yongping Zhang, Zuhua Wang, Jian Xu, Fangfang Yang, Chuanyang Dai, Weijie Xie, Zhu Liang, Songbo Su

**Affiliations:** College of Pharmaceutical Sciences, Guizhou University of Traditional Chinese Medicine, Huaxi University Town, Dongqing South Road, Guiyang, 550025 Guizhou People’s Republic of China

**Keywords:** *Corydalis yanhusuo W.T. Wang*, Quality marker, Tetrahydropalmatine, Macroreticular resin

## Abstract

*Corydalis yanhusuo W.T. Wang* alkaloids are mainly divided into three categories: protoberberine, prototropine and aporphine alkaloids. Therefore, we have taken into account these three alkaloid contents when extracting and purifying crude drugs, which is essential for the quality control of *C. yanhusuo* and its derivative products. Herein, we investigated the feasibility of the Q-marker uniform design method in the optimization of the extraction and purification of *C. yanhusuo*. In this study, Q-marker-based comprehensive scoring (CS) and uniform design methods were used to optimize the extraction and purification of *C. yanhusuo*. The inspective factors included the solvent concentration, pH, liquid–solid ratio, extraction time and frequency. Then 8 Q-markers, the total alkaloid extraction and the extraction rate were considered as the evaluating indicators during the process. The results indicated that the optimal reflux extraction process of *C. yanhusuo* was as follows: a total amount of 20 times 70% ethanol (pH = 10 of diluted ammonia), heating and refluxing twice, and extracting each time for 60 min. The results of nine-resin screening exhibited that NKA-9 macroporous adsorption resin had the best separation and purification effect on 8 kinds of *C. yanhusuo* alkaloids with stronger enrichment. During the optimal enrichment process and elution conditions, the water-soluble impurities were washed off with 5 BV distilled water at a volume flow rate of 2 BV/h, and the elution solvent was 70% ethanol with an elution volume flow rate of 1.5 BV/h and an elution dosage of 12 BV. Additionally, the total alkaloids of the obtained product were over 50%, of which eight quality markers were (+)-corydaline 3.55%, tetrahydropalmatine 3.13%, coptisine 2.79%, palmatine hydrochloride 2.24%, dehydrocorydaline 13.11%, (R)-(+)-corypalmine 2.37%, protopine 2.71% and glaucine 14.03%. Our data demonstrated that the optimal extraction and purification process was stable and feasible, which was expected to provide an experimental basis and reference for the industrial production of *C. yanhusuo*.

## Introduction

*Corydalis yanhusuo W.T. Wang* is a species in the *Corydalis* genus, and its tuber has been used as a Traditional Chinese Medicine. *C. yanhusuo* has a warm nature (Chinese medicine theory) and a pungent and bitter flavor. As an important therapeutic agent, *C. yanhusuo* can affect blood circulation promotion and air flow in the body [1]. Therefore, it is used to treat chest/flank pain, abdominal pain, chest pain, heartache, menstrual pain, postpartum stagnation and swelling [2, 3]. Previous studies have demonstrated that the main pharmacodynamic substance of *C. yanhusuo* is alkaloids, which have opioid analgesic effects, as well as antitumor [4], anti-inflammatory [5], detoxification [6], antibacterial [7] and other pharmacological effects. Most relative references, which focused on the extraction and purification process of total alkaloids from *C. yanhusuo*, were based on the single component of tetrahydropalmatine or dehydrocorydaline and used total alkaloids as the process indicators [8]. The alkaloids in *C. yanhusuo* are mainly divided into three types: the original berberine alkaloids (tertiary amine fumarate, tetrahydropalmatine, corypalmine, quaternary ammonium berberine, dehydrocorydaline and palmatine hydrochloride), the original tropine alkaloids (protopine) and the aporphine alkaloids (d-glaucine, etc.). Consequently, we must consider the content of these three components during the processes of drug extraction and total alkali purification, which is significant for quality control of *C. yanhusuo* medicinal materials and their Traditional Chinese Medicine (TCM) products.

The quality marker (Q-marker) in a TCM is a chemical substance with complex chemical structures, that are produced during the processing and preparation of TCM and TCM derived products (crude drug, decoction of TCM, extract of TCM, Chinese patent medicine preparation, etc.) [9, 10]. The Q-marker of a TCM is closely related to its functional properties and reflects its safety and effectiveness [11]. Previous studies have shown that the main pharmacodynamic substances (i.e., Q-markers) of *C. yanhusuo* are 7 alkaloids including protopine, coptisine, palmatine hydrochloride, dehydrocorydaline, (R)-(+)-corypalmine, tetrahydropalmatine and (+)-corydaline [12]. Zhang Y et al. confirmed that glaucine has a good effect on chronic pain without drug resistance [13]. In this study, we used the content of 8 kinds of *C. yanhusuo* Q-markers as research objects to investigate the extraction and purification conditions of total alkaloids from *C. yanhusuo*. The aim was to establish a simple and easy method to control extraction and purification.

## Materials and methods

### Materials

*Corydalis yanhusuo W.T. Wang* (Chinese herbs pieces, batch number: 170801, Anhui Yishengyuan Pharmaceutical Co., Ltd.). Protopine (batch number: Z26A7S13809), Tetrahydropalmatine (> 98%, batch number: Y21S7Y17091), Coptisine (batch number: P13M8F31406), Palmatine hydrochloride (batch number: Z12J7X15968), dehydrocorydaline (batch number: X11M8L35767), glaucine (batch number: W13M8Z31183), (R)-(+)-corypalmine (batch number: S10M8D35681), (+)-corydaline (batch number: Z15NTB24832), the above reference substance purity is greater than 98%, purchased from Shanghai Yuanye Biotechnology Co., Ltd. Acetonitrile is imported chromatographically pure, triethylamine is chromatographically pure, Watson’s distilled water, and the remaining reagents are of analytical grade. Zonkia HC-2062 high speed centrifuge (Anhui USTC Zonkia Scientific Instruments Co., Ltd.), Agilent 1290 infinity II (Agilent Technologies, USA), Waters UPLC (Waters, USA), ZORBAX Eclipse Plus C18 Rapid Resolution HD 2.1 × 50 mm 1.8-Micron (Agilent Technologies, USA), benchtop high speed refrigerated centrifuge (Thermo ST16R, USA), constant temperature oscillation tank (Agilent EFFU-DKZ-1).

## Methods

### Content determination of 8 Q-markers in *C. yanhusuo*

#### Chromatographic conditions

The column was ZORBAX Eclipse Plus C18 Rapid Resolution HD column (50 mm × 2.1 mm, 1.8-Micron). The mobile phase was acetonitrile (A)-0.2% glacial acetic acid (triethylamine adjusted to pH 6.0) (B), gradient elution: 0–11.5 min, 10% ~ 16% (A); 11.5–15.0 min, 16–35% (A) column; 15.0–20.0 min, 35–45%; 20–21 min, 45–58% (A); 21–22 min, 58–63 (A); 22–22.5 min, 63–95% (A). The detection wavelength was set at 280 nm. The injection volume was 2 μL. The volume Flowrate was 0.3 mL/min. The column temperature was set at 45 °C.

#### Preparation of mixed reference solution

The appropriate amounts of the protopine, coptisine, palmatine hydrochloride, glaucine, dehydrocorydaline, (R)-(+)-corypalmine, tetrahydropalmatine and (+)-corydaline reference substances were accurately weighed and dissolved in methanol to obtain a 25 mL mixed reference solution. The final concentrations of the reference substances were 95.60 μg/mL, 118.8 μg/mL, 65.60 μg/mL, 83.20 μg/mL, 90.00 μg/mL, 93.60 μg/mL, 124.8 μg/mL and 128.8 μg/mL.

#### Preparation of the test solution

The fine powder was obtained by low temperature decompression drying under various processing conditions, and after being precisely weighed, it was dissolved in methanol with ultrasonic treatment to obtain 25 mL of test solution.

#### Investigation of the linear ranges

Six reference [protopine, coptisine, palmatine hydrochloride, glaucine, dehydrocorydaline, (R)-(+)-corypalmine, tetrahydropalmatine and (+)-corydaline reference stock solutions] solutions with different concentrations were prepared by accurate drawing and measured according to the above chromatographic conditions. The peak area of the chromatogram was recorded, and the regression equation was obtained by using the peak area value as the ordinate (Y) and the amount of the reference substance as the abscissa (X).

### The content determination of total alkaloids in *C. yanhusuo*

#### Preparation of the test solution

The extracts were taken under different processing conditions, filtered and then diluted to 200 mL. Then, 1 mL was placed into a volumetric flask, and 0.5 mL 5% H_2_SO_4_ was added to make 10 mL with the pH 4.0 buffer. Then, 1 mL of the H_2_SO4-extract solution was placed into a 100 mL separatory funnel and 4.0 mL of pH 4.0 disodium hydrogen phosphate-citrate buffer solution and 7.0 mL of bromocresol green acid dye solution were added. The solution was mixed evenly, 10 activated silica gel and 5 mL of chloroform were added for 1 min; the solution was held for 5 min, and then the lower layer solution was removed to obtain the test solution.

#### Investigation of the linear ranges

The tetrahydropalmatine reference solutions were accurately transferred (0.2, 0.4, 0.6, 0.8, 1.0, 1.2 and 1.4 mL, C = 0.509 mg/mL) to volumetric flasks and then diluted with buffer solution to volume. Each reference solution was taken and processed according to the previously mentioned chromatographic conditions. The absorption value was measured at λ = 417 nm. The linear regression C of the tetrahydropalmatine was considered the X-axis, and the absorption value A was the Y-axis.

#### Comprehensive assignment of evaluation indexes

In this study, the comprehensive scoring (CS) method was used to evaluate the assignments, in which total alkaloids, the paste-forming rate and 8 Q-markers were used as indicators. Total evaluation (Y) = total content of *C. yanhusuo*/maximum content × 0.35 + eight Q-markers content M/highest content M × 0.45 + creaming rate/highest creaming rate × 0.25. The total evaluation (M) of the 8 Q-markers was calculated by the mass fraction weight coefficient method [14].

M = protopine × 0.05 + coptisine × 0.09 + palmatine hydrochloride × 0.07 + glaucine × 0.12 + dehydrocorydaline × 0.36 + (R)-(+)-corypalmine × 0.06 + tetrahydropalmatine × 0.14 + (+)-corydaline × 0.10.

### Extraction technology studies

#### Uniform design experimentation

Ten grams of *C. yanhusuo* powder (50-mesh) was weighed and transferred into an extraction vial. The solvent pH value (X1), liquid–solid ratio (X2), time/min (X3), extraction solvent ethanol concentration V/V (X4), and number of extractions (X5) were used as the main factors. With the U9 (95) uniform design method, total alkaloids (Y1), 8 Q-marker content (Y2) and paste-forming rate (Y3) were used as the evaluation indexes to optimize the alcohol extraction process of *C. yanhusuo*.

#### Verification experiment

Triplicate *C. yanhusuo* coarse powder weighing 10 g was extracted according to the optimal process selected by uniform design, concentrated in a water bath, and dried under vacuum at 60 °C under reduced pressure. The final samples were examined by UPLC.

### Purification technology studies

#### Preparation of sample solution

The total alkaloids of *C. yanhusuo* were extracted according to the above optimal extraction method. Briefly, 500 g of *C. yanhusuo* coarse powder (50 mesh) was weighed, 20 times 70% ethanol (pH 10 of diluted ammonia) was added, refluxed twice, each time for 60 min, and filtered. The filtrate was mixed three times, and ethanol was recovered, diluted with water to 1000 mL (equivalent to 0.5 g/mL of the original drug) and set aside.

#### Pretreatment of macroporous resin

A suitable amount of macroporous resin was washed with 95% ethanol solution and then the bubbles were removed through soaking and stirring to fully replace them. After resting overnight, the column was packed by the wet packing method. First, the cells were washed with 5 BV ethanol (95%) at 1 BV/h and then washed with water until all ethanol was removed. Second, the column was soaked with 5% hydrochloric acid and 5% sodium hydroxide solution for 6 h and washed with water until the pH reached neutral. Finally, 95% ethanol was used to wash the column until the effluent was turbid with water and set aside for further use.

### Resin selection

#### Determination of static adsorption rate and analytical rate

Based on the adsorption index and resolution rate of 8 kinds of *C. yanhusuo* Q-markers and total alkaloids, the appropriate types were selected from the following 9 kinds of macroporous resins. One gram of each of the pretreated 9 types of macroporous resin (AB-8, D101, DM130, HPD600, HPD100, NKA-II, NKA-9, S-8, and X-5) was placed into a conical flask. A *C. yanhusuo* sample solution of 25 mL was added and shaken for 24 h in a thermostatic shaker. After achieving adsorption equilibrium, the liquid was loaded to detect the contents. The macroporous resin was dried and transferred to an Erlenmeyer flask and then 50 mL of 70% ethanol was added for desorption. After 24 h, the mass concentration of eight Q-markers in the analytical solution was determined, and the measurement results were evaluated by the CS method. The adsorption rate (AR) and desolation rate (DR) of eight Q-markers were calculated according to the following formula (AR = (C_0_ − C_1_)V_1_/C_0_V_1_, DR = C_2_V_2_/(C_0_ − C_1_)V_1_)) and CS.

#### Static adsorption kinetics curve

Each NKA-9, HPD600 and D101 sample was accurately weighed to 1 g and placed in a conical flask. Then, 25 mL of loading buffer was added and transferred to the flasks in a shaking water bath (200 r/min) at 37 °C. The mass concentrations of the 8 Q-markers were measured at 0, 0.5, 1, 3, 5, 7, 9, 12 and 24 h. The adsorption kinetics of each macroporous resin was plotted as the adsorbed time (t) and the comprehensive adsorption amount Q. The above three kinds of statically adsorbed macroporous resins were drained, precisely desorbed by adding 50 mL of 70% ethanol, placed in a constant temperature incubator and shaken. The mass concentrations of the eight components in the analytical process were measured at 0, 0.5, 1, 3, 5, 7, 9, 12 and 24 h. The desorption kinetics of each macroporous resin was plotted according to desorption time (t) and the comprehensive desorption amount Q.

#### Drawing and determination of the leakage curve and the sampling amount

Pretreated NKA-9 macroporous adsorption resin (20 mL) was loaded into the column by the wet method while regulating the loading flow rate to 2 BV/h. When the volume reached 10 mL, it was collected as one part. The total amount was 22. The mass concentration of 8 kinds of Q-markers was measured in each leakage liquid. The leakage curve was plotted according to the mass concentration of 8 kinds of index components as the abscissa and the sampling amount as the ordinate.

#### The diameter to height ratio studies

The treated NKA-9 macroporous adsorption resin was packed by the wet packing method according to diameter to height ratios of 1:6, 1:8 and 1:10. Then, 30 mL of total alkaloid extract of *C. yanhusuo* was added separately and sampling was carried out according to the volume flow rate of 2 BV/h. After the sample was fully adsorbed, it was washed with 4 BV distilled water. The residual liquid was combined with distilled water. The mass concentration of the eight Q-markers was measured to calculate the adsorption rate.

#### Plotting of gradient elution curve

Since impurities (such as sugars and pigments) were present in the extraction, the impurity removal treatment was first performed. The column of adsorbed saturated NKA-9 resin was washed with 5 BV distilled water, and then gradient eluted with 5 BV of different ethanol concentrations of 10%, 30%, 50%, 70% and 90%. The eluant was collected in stages and determined to evaluate the elution effect on protopine, coptisine, palmatine hydrochloride, glaucine, dehydrocorydaline, (R)-(+)-corypalmine, tetrahydropalmatine and (+)-corydaline during the elution process. The amount of total alkaloids was calculated by M = CV, where C is the mass concentration of the 8 alkaloids in the eluent (mg/mL) and V is the amount of eluting solvent (mL); the ethanol concentration-elution was plotted.

#### Elution volume flow and terminus

The pretreated NKA-9 macroporous resin was packed in a diameter to height ratio of 1:8 by the wet packing method and then added to the sample solution for adsorption. After 5 BV of distilled water was taken to remove impurities, it was eluted with 30 BV 70% ethanol at 1.5, 2, and 3 BV/h. Each of the resin bed eluents was collected to determine and calculate the total alkaloid content and the cumulative elution amount. The elution amount ${\text{Q}}\left( \% \right) = \left( {\sum\nolimits_{\text{i = 1}}^{ 2 0} {\left( {\text{mi + mi + 1}} \right)} /{\text{M}}} \right) \times 100\%$, where mi is the amount of 8 alkaloids in the part i eluent, and M is the amount of 8 alkaloids in the loading solution.

#### Determination of water consumption for flushing impurities

The pretreated NKA-9 resin was packed at a diameter to height ratio of 1:8, and then the sample solution was added and eluted with 3, 5, 7, 9 and 12 BV distilled water. A mold reaction was used to monitor the washing of impurities such as sugar. A negative Molish’s test indicated that the elution of impurities was complete. To monitor the Molish reaction, the sample was further eluted with 12 BV 70% ethanol at 1.5 BV/h. The paste-forming and comprehensive loss rates of the eight alkaloids were determined and calculated.

#### Verification of the purification process

Through the above investigations, the conditions of the purification process were as follows: NKA-9 macroporous adsorption resin with a column diameter ratio of 1:8; loading volume of 6 BV, water washing of 5 BV; 70% ethanol 12 BV elution; and flow rate of 1.5 BV/h. To further investigate the feasibility and stability of the optimal process for the verification test, a total of 450 mL of the sample solution was added to a column containing 150 mL of NKA-9 resin, and then the operation was carried out according to the process conditions. Finally, the ethanol eluate was collected and recovered by vacuum drying at 60 °C. UPLC and UV were used to measure and determine the content of 8 Q-markers in the product and the amount of total alkaloid, respectively. Furthermore, in consideration of the inherent drawbacks (below industrial production) of bench-scale research, a preliminary scale-up verification test was also carried out.

## Results

### Content determination of 8 Q-markers in *C. yanhusuo*

**Fig. 1:**
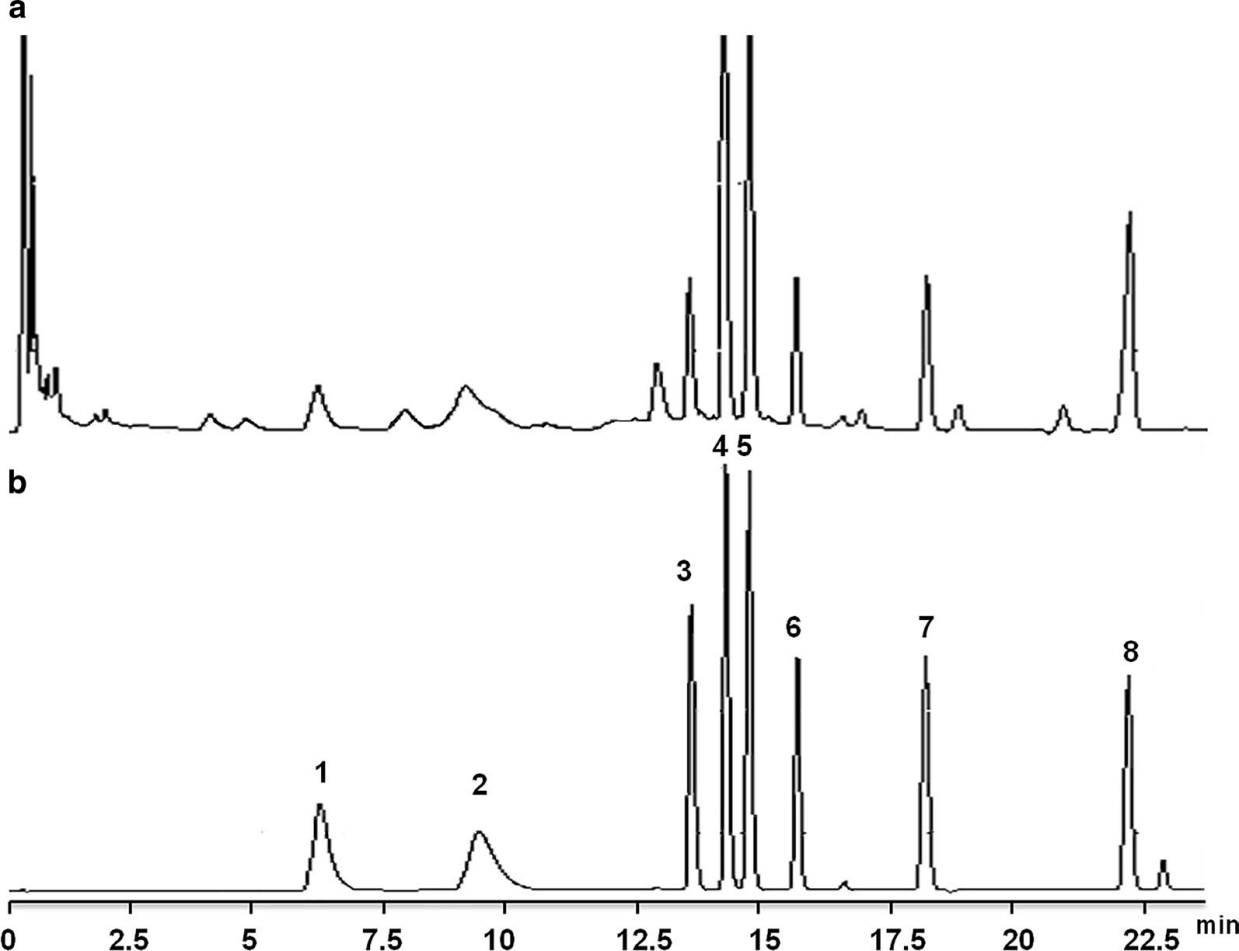
The UPLC chromatogram of 8 Q-markers, each peak represents protopine, coptisine, palmatine hydrochloride, glaucine, dehydrocorydaline, (R)-(+)-corypalmine, tetrahydropalmatine and (+)-corydaline from 1 to 8, respectively. **a** The samples chromatogram; **b** the chromatogram of reference substances

It is important to establish a simultaneous quantitative chromatography analysis method of TCM for optimizing the extraction and purification of *C. yanhusuo.* Under the above chromatographic conditions, the samples were further analyzed by UPLC to obtain the chromatogram between the samples and reference substances. In this study, the number of theoretical plates was no less than 5000 per component. As shown in Fig. 1b, the peaks in chromatogram of the mixed references could be observed clearly and separated well, in which each peak represents protopine, coptisine, palmatine hydrochloride, glaucine, dehydrocorydaline, (R)-(+)-corypalmine, tetrahydropalmatine and (+)-corydaline from 1 to 8. In addition, the peaks in the chromatogram of the samples were basically consistent with the peaks in the chromatogram of the mixed references (Fig. 1a), which indicated that the eight components could be well separated from *C. yanhusuo* by UPLC.

### Investigation of the linear ranges

To ensure the method accuracy, the linear ranges of eight reference substances were further investigated. The regression equations and linear ranges of the protopine, coptisine, palmatine hydrochloride, glaucine, dehydrocorydaline, (R)-(+)-corypalmine, tetrahydropalmatine and (+)-corydaline reference stock solutions were Y = 4757.5X − 4.304 (r = 0.9999) and 0.0096–0.2100 μg; Y = 4349.7X − 6.606 (r = 0.9998) and 0.0112–0.2610 μg; Y = 7927.2X − 4.130 (r = 0.9998) and 0.0066–0.1441 μg; Y = 8331.4X − 1.378 (r = 0.9998) and 0.0083–0.1834 μg; Y = 7792.6X − 1.872 (r = 0.9999) and 0.0090–0.1983 μg; Y = 4151.7X − 4.248 (r = 0.9998) and 0.0093–1.1871 μg; Y = 4078.7X − 4.883 (r = 0.9998) and 0.0124–0.2508 μg; Y = 3677.3X − 5.653 (r = 0.9998) and 0.0131–0.2586 μg, respectively. The results showed that the linear correlation of each index component fit well in the linear range.

For the content determination of total alkaloids in *C. yanhusuo*, extractions through different process conditions were prepared, and the linear range of tetrahydropalmatine was also investigated. Finally, the linear regression equation Y = 8.993X − 0.0539 (R = 0.9996) was obtained by data analysis and linear fit. The results showed that the standard curve had good linear correlation in the linear range of 0.020–0.143 mg/mL.

### Study on the extraction process

Total alkaloid (Y1), 8 Q-marker content (Y2) and creaming rate (Y3) were used as the evaluation indexes by the U9 (95) uniform design method to optimize the alcohol extraction process of *C. yanhusuo*.

As shown, the data in Table 1 were recorded into the DPS data processing system of Zhejiang University. The step-by-step linear regression method was used to obtain the multiple linear regression equation, Y = 11.814 + 0.151X_1_ + 2.164X_2_ + 0.336X_4_ + 9.290X_5_, R = 0.951, F = 9.214 (*P *= 0.026 < 0.05). The results indicated that the model correlation is good and can be applied to the fitting of the test data. From the equation result it is known that the main factors affecting the absorbance are solvent pH, liquid–solid ratio, solvent concentration and number of extractions. The best conditions obtained by the equation are X_1_ = 10, X_2_ = 20, X_3_ = 60, X_4_ = 70% and X_5_ = 2. Therefore, the optimal extraction scheme of total alkaloids of *C. yanhusuo* was adding 20 times ethanol (pH = 10, 70%) as the extraction solvent twice, each time for 60 min. With the extraction scheme, *C. yanhusuo* could be extracted completely. To validate the feasibility of the U9 (95) uniform design method, the total alkaloid extractions were prepared from *C. yanhusuo* according to the optimal process selected by uniform design. The results showed that total alkaloids of *C. yanhusuo*, the amount of 8 Q-markers and the rate of ointment were 18.82 mg/g, 7.59 mg/g, and 22.92%, respectively, which indicated that the extraction process conditions obtained by the uniform test were stable and reliable.

**Table 1:** Uniform design table and its results (n = 3)

No	X1	X2	X3/min	X4 (V/V)	X5	Y1 (mg/g)	Y2 (mg/g)	Y3 (%)	Y
N1	10	16	105	50	3	17.30	7.88	15.66	13.52
N2	7	6	120	20	3	13.59	5.72	15.93	11.31
N3	4	4	90	60	1	10.96	4.88	6.24	7.59
N4	2	10	150	40	2	15.77	6.80	27.47	15.45
N5	5	20	75	10	2	18.13	6.99	19.44	14.35
N6	6	18	135	80	1	18.34	7.26	6.43	11.29
N7	8	12	30	30	1	15.40	5.24	12.59	10.90
N8	3	14	45	70	3	15.80	7.62	12.73	12.14
N9	9	8	60	90	2	14.79	6.76	5.59	9.62

### Study on the purification process

To investigate the purification technology of *C. yanhusuo*, 9 types of macroporous resin, AB-8, D101, DM130, HPD600, HPD100, NKA-II, NKA-9, S-8 and X-5, were selected and studied. The results are listed in Table 2.

**Table 2:** AR and DR of different type of macroporous resins at 8 Q-markers

Resin type	NKA-II	NKA-9	HPD600	HPD-100	D101	DM130	AB-8	S-8	X-5
Coptisine									
AR/%	77.67	90.97	88.81	82.86	86.50	82.34	71.54	83.76	74.25
DR/%	42.13	55.43	53.27	47.32	50.96	46.80	36.00	48.22	38.71
Protopine									
AR/%	73.12	86.42	84.26	78.31	81.95	77.79	66.99	79.21	69.70
DR/%	39.67	52.97	50.81	44.86	48.50	44.34	33.54	45.76	36.25
Dehydrocorydaline									
AR/%	69.23	82.53	80.37	74.42	78.06	73.90	63.10	75.32	71.34
DR/%	37.89	51.19	49.03	43.08	46.72	42.56	31.76	43.98	40.00
Palmatine hydrochloride									
AR/%	80.14	93.44	91.28	85.33	90.97	84.81	74.01	86.23	82.25
DR/%	35.65	48.95	46.79	40.84	46.48	40.32	29.52	41.74	37.76
Tetrahydropalmatine									
AR/%	80.45	93.75	91.59	88.34	91.28	85.12	74.32	86.54	74.61
DR/%	45.67	58.97	56.81	52.76	56.50	50.34	39.54	51.76	30.12
(+)-corydaline									
AR/%	76.45	89.75	87.59	83.54	88.72	81.12	70.32	82.54	74.92
DR/%	45.15	58.45	56.29	52.24	57.42	49.82	39.02	51.24	40.14
(R)-(+)-corypalmine									
AR/%	76.67	89.97	87.81	83.76	88.94	81.34	70.54	82.76	73.01
DR/%	42.86	56.16	54.00	49.95	55.13	47.53	36.73	48.95	28.52
Glaucine									
AR/%	78.24	91.54	89.38	85.33	90.51	82.91	72.11	84.33	73.32
DR/%	42.62	55.92	53.76	49.71	54.89	47.29	36.49	48.71	38.54
CS									
AR/%	75.94	89.10	86.97	81.69	86.35	80.56	69.87	81.97	75.30
DR/%	39.18	52.35	50.21	44.89	49.59	43.81	33.11	45.21	36.41

It is known that three types of macroporous resins (NKA-9, HPD600 and D101) have good effects on the adsorption rate and resolution rate of the 8 kinds of *C. yanhusuo* Q-markers (Table 2). Therefore, based on the static adsorption kinetics curve, the purification effects of NKA-9, HPD600 and D101 3 macroporous resins on total alkaloids of *C. yanhusuo* are worthy of further investigation.

**Fig. 2:**
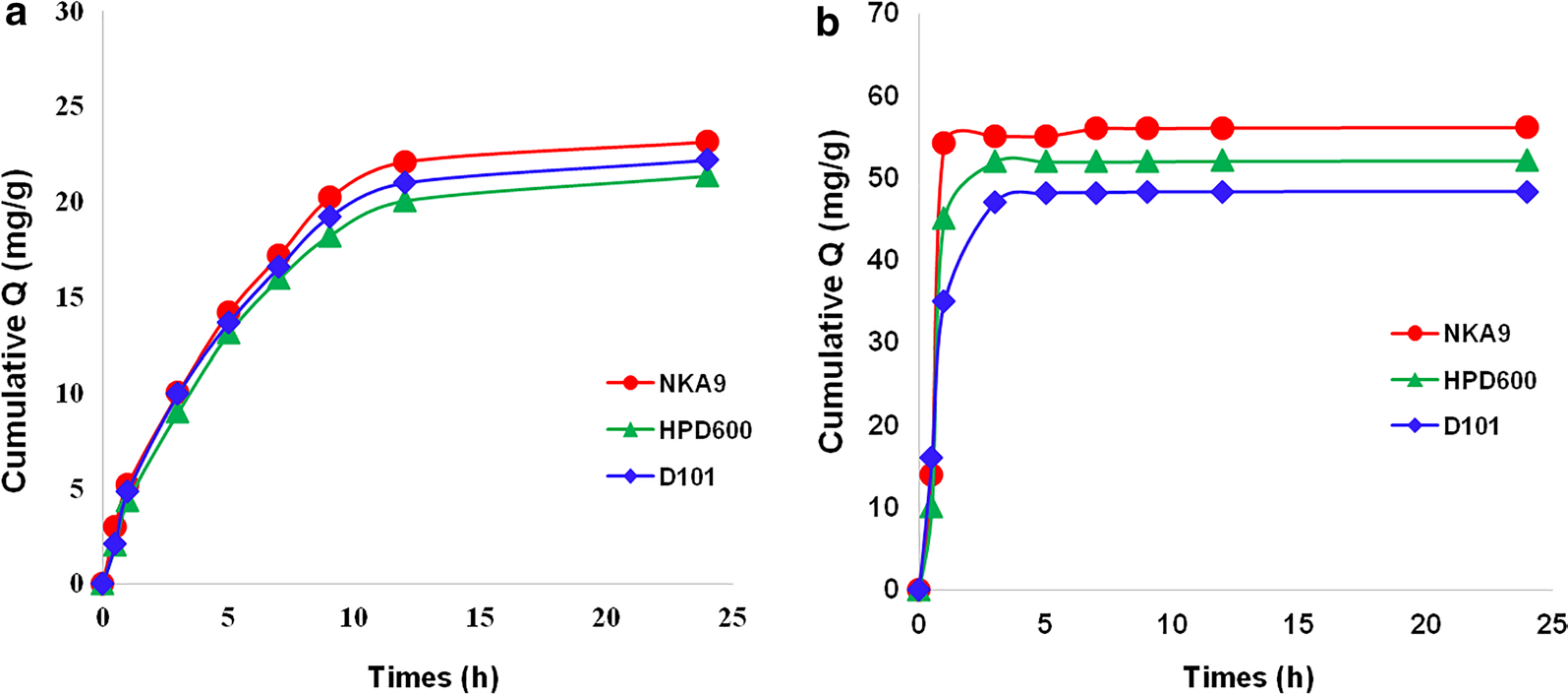
The kinetics curves of each macroporous resin to 8 kinds of *C. yanhusuo* Q-markers. **a** Adsorption curves and **b** desorption curves

The adsorption and desorption kinetics of each macroporous resin are shown in Fig. 2.

Figure 2a shows that the adsorption effect of NKA-9 to the 8 kinds of *C. yanhusuo* Q-markers was better than HPD600 and D101, which was preferable for separating the 8 kinds of *C. yanhusuo* Q-markers. In addition, NKA-9 reached an adsorption equilibrium at approximately 12 h and complete desorption at approximately 4 h (Fig. 2b). Therefore, NKA-9 was eventually considered the best macroporous adsorption resin in our further studies.

**Fig. 3:**
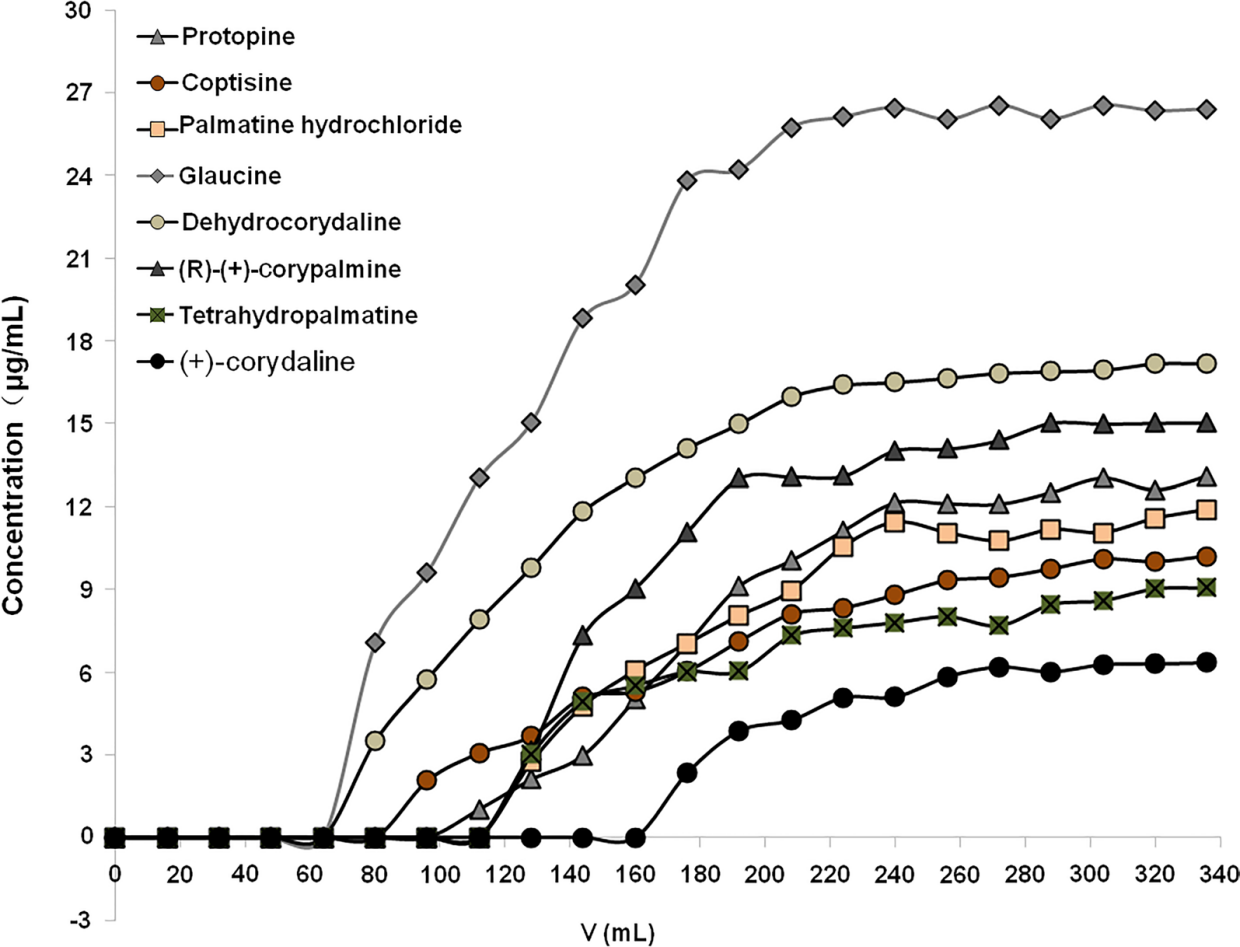
The leakage curves of protopine, coptisine, palmatine hydrochloride, glaucine, dehydrocorydaline, (R)-(+)-corypalmine, tetrahydropalmatine and (+)-corydaline

The leakage curves of the 8 index components are shown in Fig. 3. Glaucine and dehydrocorydaline started to leak when the sampling amount was 60 mL and reached saturation at 200 mL. Coptisine began to leak at 100 mL, and the adsorption reached saturation at 240 mL. Protopine, (R)-(+)-corypalmine and tetrahydropalmatine began to leak at 120 mL, and reached saturation at 240 mL. (+)-corydaline leaked at 160 mL and reached saturation at 280 mL. Based on the above analyses, 60 mL was finally determined as the maximum sampling amount, which was 1.5 g raw medicine/mL resin, because the leakage of the 8 Q-markers was much less at this time.

To confirm the optimal diameter of the height ratio and calculate the adsorption rate, the mass concentration of the eight Q-markers was further measured. The results showed that the adsorption rates of the eight Q-markers increased with increasing diameter-to-height ratio (Table 3). When the diameter to height ratio increased to 1:10, the adsorption rate of macroporous resin to 8 Q-markers was close to 100.00%. However, there was no significant difference in the adsorption rate (the ratio of diameter to height) between 1:8 and 1:10; therefore, the diameter to height ratio of 1:8 was the best option for packing.

**Table 3:** The effect of NKA-9 with the different diameter to height ratio on the adsorption rate (n = 3)

D/H	AR%								
	Protopine	Coptisine	Palmatine hydrochloride	Glaucine	Dehydrocorydaline	(R)-(+)-corypalmine	Tetrahydropalmatine	(+)-corydaline	CS
1:6	94.25	96.05	63.41	99.25	89.62	87.35	89.21	87.32	89.95
1:8	98.35	99.24	95.35	100	100	100	100	98.32	98.24
1:10	99.23	100	98.24	100	100	100	100	100	98.75

**Fig. 4:**
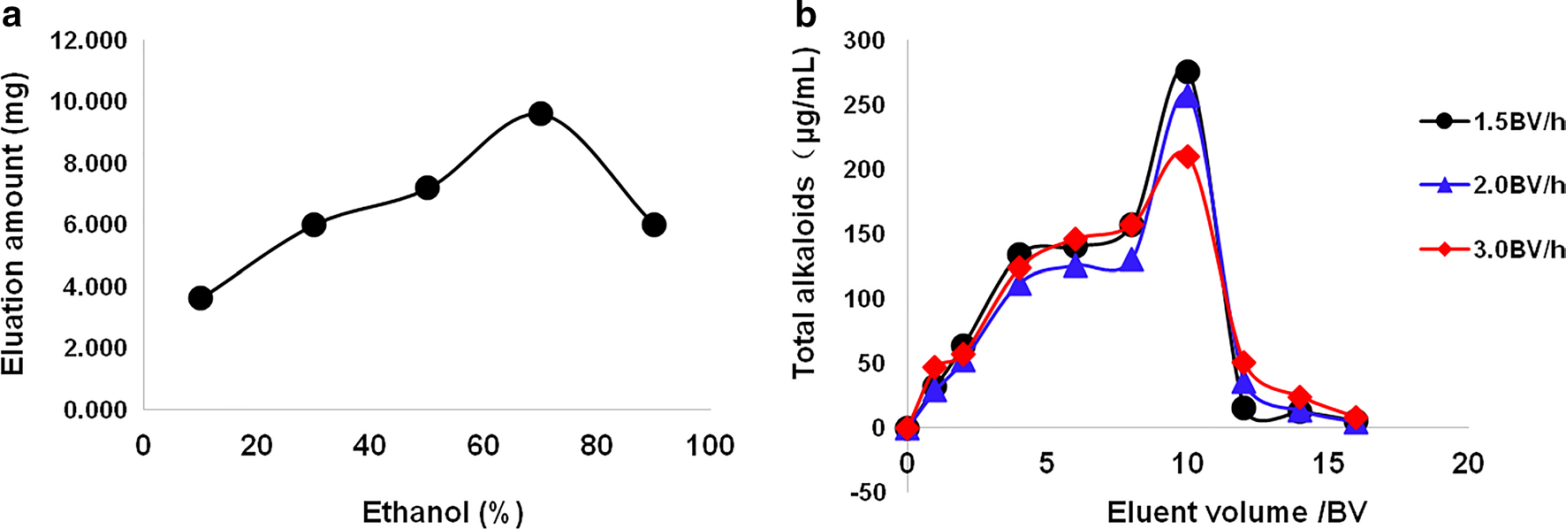
Effects of different ethanol concentration and volume flow on eight Q-markers of *C. yanhusuo*. **a** ethanol concentration and **b** eluent volume

In addition, the ethanol concentration-elution chart was shown in Fig. 4a. We found that the eight Q-markers were the highest when obtained from the eluent with 70% ethanol. The results also showed that 8 Q-markers were almost eluted when the elution flow rate was 1.5 BV/h and the amount of eluent was 12 BV (Fig. 4b).

The amount of impurifying water was determined as well. Table 4 shows that alkaloids had different degrees of loss when using distilled water as an impurity eluent, which increased as the amount of water increased. When the elution water consumption increased from 5 to 7 BV, the Molish reaction was negative, but the total alkaloid loss rate increased from 0.43 to 2.36%. Therefore, 5 BV distilled water was finally selected as the impurity elution volume.

**Table 4:** Investigation of water consumption for flushing impurities (n = 3)

The elution quantity (BV)	The loss rate of the eight alkaloids/%	The quantity of paste/mg	Alkaloids in paste/%
3	0.11	145.56	1.21
5	0.43	89.23	2.56
7	2.36	78.35	4.77
9	3.22	75.53	6.03
12	6.21	70.17	8.28

To verify the optimal purification process and investigate its feasibility and stability, we carried out a verification test. The results showed that the total alkaloids of the obtained products reached more than 50%, among which the 8 kinds of quality markers were 3.55% (+)-corydaline, 3.13% tetrahydropalmatine, 2.79% coptisine, 2.24% palmatine hydrochloride, 13.11% dehydrocorydaline, 2.37% (R)-(+)-corypalmine, 2.71% protopine and 14.03% glaucine. All of these results indicated that it is feasible to optimize the extraction and purification process of *C. yanhusuo* by the Q-marker uniform design method. The results also proved that the data obtained from the Q-marker uniform design method were reliable. In addition, regarding the scale-up verification test, we had already obtained 15X scale-up test data. Our initial results showed that the total alkaloid contents were more than 50% when 3.4 kg (15X scale-up) *C. yanhusuo* powder was extracted and purified by the optimized conditions. Moreover, the eight Q-markers of the total alkaloids were (+)-corydaline 3.51%, tetrahydropalmatine 2.88%, coptisine 2.44%, palmatine hydrochloride 2.21%, dehydrocorydaline 12.68%, (R)-(+)-corypalmine 2.21%, protopine 2.52%, and glaucine 13.54%. The pilot-scale test results were the same as the bench-scale research results, with some components showing a downward trend, which is consistent with the trend of the leak curve study results.

## Discussion

Extraction is an indispensable process of producing Chinese medicinal materials and their products, but the final result could be affected by various factors. In addition to the conventional extraction factors, the selection of detective indicators plays an important role in the accuracy of multi-index decision-making, so it is very important to evaluate as many data indicators as possible during data processing. Previous research has shown that the main medicinal substances of *C. yanhusuo* are the total alkaloids and auxiliary medicinal ingredients (polysaccharides and organic acids). Therefore, we selected the total alkaloids, paste-forming rate and 8 Q-markers [12, 15] as indicators for the purification process.

Chinese medicine Q-markers are used as an indicative substance to reflect the safety and effectiveness of TCM. Therefore, the quality of a TCM could be better controlled if drug-inherent Q-markers are reasonably selected during the extraction process. Zhang Tiejun et al. [12] confirmed that the Q-markers of *C. yanhusuo* are mainly 7 alkaloids including (+)-corydaline, tetrahydropalmatine, coptisine, palmatine hydrochloride, dehydrocorydaline, (R)-(+)-corypalmine and protopine. Hence, this study has rationality in the selection of indicators and can fully reflect the quality of *C. yanhusuo*.

There is a certain amount-to-quantity relationship among the components in Traditional Chinese Medicine, and their contributions to the efficacy are different. Thus, the weight coefficients of each component in the overall component quality are different. To reduce the computational complexity of data processing, the mass fraction weight coefficient method [14, 16] was used as an evaluation index and given a higher weight. In this study, we fully considered the complexity of the chemical composition and the diversity of the pharmacodynamic substance basis in the selection of the indicators and the weights of the indicators. In addition, the mass fraction weight coefficient method was used to process the multicomponent data and fully considered the relationship of *C. yanhusuo* decoction pieces between the intrinsic chemical constituents of total alkaloids. On this basis we established a simultaneous quantitative chromatography analysis method of TCM, which laid the foundation for further optimizing the extraction and purification of *C. yanhusuo* (Fig. 1). To insure the method accuracy, the linear ranges of the eight reference substances were further investigated. The results showed that the linear correlation of each index component was fitted well in the linear range, and the standard curve also had good linear correlation in the linear range of 0.020–0.143 mg/mL.

Reflux is commonly used for extraction. According to the literature, the pH value of the extraction solvent could affect the dissolution of alkaloids [17]. It was found by the correlation factor investigation that the extraction of total alkaloids from *C. yanhusuo* was affected by the pH value of the solvent, the total alkali content was increased with the increase of the solvent, and the equilibrium concentration pH value existed. Therefore, on the basis of the comprehensive scoring (CS) method, it is significant to optimize the alcohol extraction process of *C. yanhusuo* by the U9 (95) uniform design method. In this study, total alkaloid (Y1), 8 Q-marker content (Y2) and paste-forming rate (Y3) were selected as the evaluation indexes. The multiple linear regression equation results indicated that the model correlation is good and can be applied to the fitting of the test data. In addition, further results showed that the main factors affecting the absorbance are solvent pH, liquid–solid ratio, solvent concentration and number of extractions. Using U9 (95) uniform design method, the optimal extraction process of total alkaloids of *C. yanhusuo* was determined and demonstrated by the verified experiment, which indicated that the extraction process conditions obtained by the uniform test were stable and reliable.

During the purification process, we chose the macroporous resin as the impurity removal material based on previous studies [18–20], and nine resins with different polarity were investigated in the screening test.

The adsorption–desorption abilities, static and dynamic adsorptive capacity, water consumption of flushing impurities and diameter to height ratio are the important indicators of the performance and separation characteristics of macroporous resin. To select the optimal purifying process of the macroporous resin more objectively and effectively, we systematically investigated 9 types of macroporous resin, AB-8, D101, DM130, HPD600, HPD100, NKA-II, NKA-9, S-8 and X-5. Since the water content of each resin was different, the final result was converted into the component adsorption or desorption amount per gram of dry resin. Our data confirmed that NKA-9 simultaneously had good adsorption and desorption abilities and static and dynamic adsorptive capacity with high resin utilization rate and low cost compared to scopolamine alkaloids (tertiary amines such as (+)-corydaline, tetrahydropalmatine, (R)-(+)-corypalmine, quaternary ammonium coptisine, dehydrocorydaline and palmatine hydrochloride), protopine alkaloids (protopine) and aporphine alkaloids (d-glaucine, etc.). In addition, under the optimized process conditions, the total alkaloid content of *C. yanhusuo* could reach over 50%. The contents of the 8 quality markers were 3.55%(+)-corydaline, 3.13% tetrahydropalmatine, 2.79% coptisine, 2.24% palmatine hydrochloride, 13.11% dehydrocorydaline, 2.37% (R)-(+)-corypalmine, 2.71% protopine and 14.03% glaucine. These parameters could meet the requirements of the effective content of new Chinese medicines.

Through the above research, we determined the purification techniques of NKA-9 macroporous adsorption resin as follows: column diameter ratio of 1:8; loading volume of 6 BV, water washing of 5 BV; 70% ethanol 12 BV elution; and flow rate of 1.5 BV/h. To further verify the feasibility and stability of the optimal process, a 15X scale-up verification test was carried out. Our initial data showed that the pilot-scale test results were the same as the bench-scale research results, with some components showing a downward trend, which was consistent with the trend of the leak curve study results. Therefore, the purification process has good practical prospects for application.

## Conclusion

To improve treatment efficacy using the least dose and to facilitate processing, the useful components from TCM should be extracted. However, the extraction and purification of TCM has been difficult, and there has been a lack of percise solutions until now. In this investigation, we employed Q-marker-based CS and uniform design methods to optimize the extraction and purification of *C. yanhusuo*. Then, the stepwise statistical method was used to evaluate the existing factors affecting the extraction and purification of *C. yanhusuo*, including the solvent concentration, pH value, liquid–solid ratio, extraction time and frequency. Then, 8 Q-markers, total alkaloid extraction and extraction rate were considered the evaluating indicators. The results indicated that the optimal extraction and purification process was stable and feasible, which is expected to provide experimental support for the industrial production of *C. yanhusuo*.

## Data Availability

The datasets used and/or analyzed in this study are available from the corresponding author upon request.

## Abbreviations

*C. yanhusuo*: *Corydalis yanhusuo W.T. Wang*
CS: Comprehensive scoring
TCM: Traditional Chinese Medicine
AR: Adsorption rate
DR: Desolation rate
